# Controlled comparative study of YOLOv8-Pose, YOLOv11-Pose, and Detectron2 for vertebrae detection and keypoint estimation

**DOI:** 10.1371/journal.pone.0347290

**Published:** 2026-04-21

**Authors:** Avinash Bansal, Jan Kubíček, Shalini Garg

**Affiliations:** Department of Cybernetics and Biomedical Engineering, VSB-Technical University of Ostrava, FEECS, Ostrava, Poruba, Czech Republic; Polytechnic Institute of Leiria: Instituto Politecnico de Leiria, PORTUGAL

## Abstract

Accurate vertebrae detection with precise keypoint localization is essential for medical image analysis and clinical applications, such as spinal alignment assessment. This study presents a controlled, task-driven comparison of four pose-based deep learning models for vertebrae detection and keypoint estimation: YOLOv8n-Pose, YOLOv11n-Pose, and Detectron2 (Keypoint R-CNN) with ResNet-50 and ResNet-101 backbones. A single-class vertebrae dataset with bounding box annotations and four anatomically meaningful keypoints per vertebra was evaluated under consistent training settings. Performance was assessed in terms of keypoint localization accuracy, detection precision, inference speed, and vertebrae-specific prediction behavior, including detection completeness and duplicate detections, which are critical for clinical usability. Results show that YOLOv11n-Pose provides the best balance between keypoint accuracy and inference efficiency, while YOLOv8n-Pose achieves the fastest inference with competitive performance. Detectron2-based models exhibit lower keypoint accuracy, slower inference, and frequent duplicate predictions that obscure correct anatomical landmarks. Additional experiments indicate that larger YOLO variants (particularly YOLOv8l-Pose) improve accuracy, albeit at increased computational cost. Overall, the results demonstrate that model capacity or architectural recency alone does not guarantee superior performance in vertebrae keypoint detection, highlighting the importance of anatomy-aware model selection for spine imaging applications.

## Introduction

Understanding the spine’s complex structure with high precision is critical for medical image analysis, supporting a wide range of clinical applications. Accurate identification of vertebral structures is vital for assessing spinal alignment, diagnosing scoliosis, evaluating degenerative spinal diseases, and planning surgical interventions such as spinal fusion and vertebroplasty [1–3]. The complexity of spinal anatomy, with its repetitive yet subtly varying vertebral shapes, presents a significant challenge for automated methods, particularly when dealing with noisy or low-contrast images.

Traditional image analysis and segmentation techniques, including thresholding, edge detection, and morphological operations, often fall short when applied to spine imaging. These methods are sensitive to imaging artifacts, noise, and anatomical variability, and they cannot typically generalize across different patients and imaging modalities [4]. Early machine learning-based methods attempted to improve performance by incorporating handcrafted features and statistical models, such as active shape models (ASMs) and support vector machines (SVMs); however, these approaches still struggled with occlusions, overlapping anatomical structures, and the variability in vertebral morphology, especially in modalities like X-ray and magnetic resonance imaging (MRI), where the contrast between bone and soft tissue can be minimal [5,6].

In recent years, deep learning has emerged as a transformative tool in medical imaging, achieving superior performance over classical approaches in tasks such as classification, detection, and segmentation. Convolutional neural networks (CNNs), particularly U-Net and its variants, have become the de facto standard for medical image segmentation [7]. More recent methods have evolved beyond voxel-wise segmentation to include object detection frameworks such as YOLO (You Only Look Once) and Faster R-CNN (Region-based Convolutional Neural Network), which can detect and localize vertebrae using bounding boxes [8].

Despite these advances, the effectiveness of modern pose estimation models for vertebrae keypoint detection remains underexplored. Pose-based models, originally developed for applications such as action recognition and motion analysis, have been adapted for medical purposes, providing a richer representation of anatomical structures. Pose-based models enable the prediction of discrete anatomical landmarks (keypoints), which offer several advantages over bounding box-based methods. In the context of vertebrae, keypoints can capture anatomical features such as the center of the vertebral body, spinous process tips, and vertebral corners, providing detailed information about orientation, rotation, and curvature [9–11]. This granularity is especially beneficial for clinical assessments that rely on precise alignment and symmetry, such as Cobb angle measurement in scoliosis or spinal curvature monitoring in degenerative diseases [12,13].

Moreover, keypoint-based models can better handle the complex spatial relationships between vertebrae in a sequence, making them well-suited for downstream tasks such as spine reconstruction and curvature analysis.

Building on these advancements, this paper evaluates and compares the effectiveness of four deep learning models for vertebrae detection with keypoint estimation (pose estimation): YOLOv8n-Pose, YOLOv11n-Pose, and Detectron2 (Keypoint R-CNN) with ResNet-50 and ResNet-101 backbones incorporating Feature Pyramid Networks (FPN). YOLOv11 (officially YOLO11), released by Ultralytics in September 2024 [14], represented the latest stable iteration of the YOLO framework available during the experimental phase of this study (conducted through March 2025). For consistency and easier comparison with YOLOv8 models, we adopt the “v” naming convention in YOLOv11n-Pose. It extends the YOLOv8 framework with refined architectural components, including an optimized backbone and neck for improved feature extraction. Key updates include more efficient Cross Stage Partial (CSP) blocks, such as C3k2 (a C3 module with two convolutional layers), and the C2PSA (Cross Stage Partial Spatial Attention) mechanism, which are designed to balance accuracy, parameter efficiency, and inference speed.

These models are evaluated on a vertebrae-specific dataset in terms of keypoint localization, detection accuracy, detection completeness, and computational efficiency, metrics that are highly relevant for real-time clinical applications.

**The key objectives of this study are:**

- To assess the ability of pose-based models to detect vertebrae and localize clinically meaningful keypoints, addressing challenges unique to spinal anatomy.
- To compare the performance trade-offs between lightweight and high-capacity models for real-time vertebrae detection scenarios.
- To identify strengths and limitations of each model in terms of robustness to keypoint visibility, accuracy of localization, and the occurrence of vertebrae-specific duplicate/missing predictions.
- To recommend the most suitable model for clinical and research scenarios where fast and accurate vertebra detection with keypoints is needed.

The remainder of this paper is organized as follows. The Related work section reviews previous studies and identifies existing gaps in vertebrae keypoint localization. The Materials and methods section details the dataset preparation, annotation formats, selected models, and training configurations employed in this study. The Results section presents quantitative and qualitative comparisons of YOLO-Pose and Detectron2 models, emphasizing accuracy, keypoint completeness, inference speed, and augmentation effects. The Discussion section highlights factors affecting performance, trade-offs between accuracy and efficiency, and study limitations with future directions. Finally, the Conclusion section summarizes the main contributions and findings of the study.

### Related work

Deep learning has significantly advanced the field of medical image segmentation, particularly for complex anatomical structures like the spine. Traditional approaches relied heavily on handcrafted features and classical image-processing techniques [2,15,16], which often lacked robustness across diverse patient populations and imaging conditions due to their dependence on manually designed features such as edge detection, intensity thresholding, or shape priors. Recent years have seen a shift toward data-driven models, especially convolutional and transformer-based architectures, which offer improved adaptability and generalizability across imaging modalities like computed tomography (CT) and MRI [4,17].

Several studies have compared mask-based segmentation models in spinal imaging tasks [18,19], but these evaluations typically focus on dense pixel-wise outputs rather than sparse, keypoint-based vertebrae localization. This gap motivates the exploration of real-time detectors for vertebrae keypoints, which are crucial for clinical applications, including surgical planning, deformity quantification, and spinal alignment assessment.

### CNN-based and U-Net architectures

Convolutional neural networks (CNNs) have dominated medical segmentation due to their hierarchical feature representation capabilities. U-Net [7], one of the most influential architectures in biomedical image segmentation, uses skip connections and multi-resolution pathways to achieve high localization accuracy with relatively few training samples. Variants like Attention U-Net [20] and V-Net [21] have improved upon this by incorporating attention modules or 3D convolutions for volumetric data. In the context of vertebrae segmentation, researchers have applied U-Net with multi-scale input [22] and shape priors [23] to enhance performance. However, these methods typically yield dense pixel-wise masks and are computationally intensive, making them less suitable for real-time or lightweight deployment scenarios.

### Keypoint-based methods

Keypoint-based approaches have emerged as an alternative for anatomical structure representation, offering sparse, interpretable outputs well-suited for downstream tasks like pose estimation and motion tracking. Notable examples include HRNet [24] and HigherHRNet [25], which excel in human pose estimation by capturing fine-grained structural cues. Some studies have adapted these methods to medical imaging, including for detecting landmarks in cephalometric radiographs [26], spinal X-rays [27], and vertebral maturation stages [28]. However, these models often struggle with anatomical variability and limited labeled data in medical domains, requiring intensive domain-specific tuning to perform effectively on medical imagery.

### Real-time object detection with pose estimation

Recent unified detection-and-pose frameworks, such as YOLOv8n-Pose, YOLOv11n-Pose, and Detectron2 with keypoint heads, enable simultaneous bounding-box detection and keypoint localization in a single forward pass [29–31]. These models provide an efficient balance between inference speed and accuracy, making them suitable for real-time or resource-limited scenarios.

While more complex architectures, including transformer-based (e.g., ViTPose [32]) and certain graph-based models, have shown superior performance in general pose estimation tasks, they typically require massive computational resources, large-scale pretraining data, and substantial memory, which limits their practicality for latency-sensitive clinical applications.

Considering the unique challenges of spine imaging, including low contrast, anatomical occlusions, and repetitive vertebral structures, this study focuses on evaluating YOLOv8n-Pose, YOLOv11n-Pose, and Detectron2 (Keypoint R-CNN with ResNet-50/101 backbones) in a controlled, task-specific comparison. These models provide the most viable trade-off for vertebrae keypoint estimation in research and clinical workflows.

### Research gap

Although several studies have compared object detection frameworks like YOLO and Detectron in general-purpose or non-medical domains [29,30], these comparisons typically involve earlier versions of the models (e.g., YOLOv3, Faster R-CNN, Detectron1) and focus primarily on classification or bounding-box detection rather than anatomical keypoint localization.

While some research has attempted to compare compression and detection performance across YOLO and Detectron frameworks, many of these works lack detailed reporting of experimental settings, hyperparameters, or evaluation metrics, making it difficult to reproduce results or assess the fairness of their comparisons. In several cases [33–35], critical configuration details are either omitted or ambiguously described, limiting their utility for benchmarking or for translation to clinical applications such as anatomical keypoint detection.

Recent advances in anatomical landmark localization [19,36,37] have demonstrated the potential of keypoint-based approaches; however, to the best of our knowledge, no prior work has systematically evaluated modern, pose-capable variants, specifically YOLOv8n-Pose, YOLOv11n-Pose, or Detectron2 with Keypoint R-CNN heads, for vertebrae keypoint detection in spinal imaging. This represents an important research gap, as the comparative performance of lightweight (nano variants) versus high-capacity models remains unexplored, leaving uncertainty regarding model suitability for real-time clinical workflows.

To address this gap, the present study benchmarks both lightweight and high-capacity pose estimation detectors for vertebrae keypoint localization, a task vital for clinical applications, including surgical planning, deformity quantification, and computer-assisted diagnosis. Our experiments compare these models using a standardized dataset and consistent training configurations, reporting results on keypoint accuracy, bounding-box precision, inference speed, and clinically relevant behaviors. These findings provide practical, actionable insights for real-time deployment in spine-focused healthcare workflows, revealing domain-specific trade-offs not fully captured in general pose estimation or prior spine segmentation studies.

## Materials and methods

This section outlines the dataset, annotation formats, selected models, and training procedures used in this study. Our goal is to establish a reproducible and fair comparison of multiple keypoint detection models applied to vertebrae localization in X-ray images.

To improve clarity and facilitate a better understanding of the technical content, Table 1 summarizes key abbreviations and terminology frequently used throughout this study.

**Table 1:** List of common abbreviations.

Abbreviation	Definition	Abbreviation	Definition
AMP	Automatic Mixed Precision	Kpts	Keypoints
BCE	Binary Cross-Entropy	L1	L1 Loss (Mean Absolute Error Formulation)
BBox	Bounding Box	mAP	Mean Average Precision
CIoU	Complete Intersection over Union	OKS	Object Keypoint Similarity
CSP	Cross Stage Partial	RoI	Region of Interest
Detectron2-R101	Detectron2 framework using ResNet-101 backbone with FPN	RPN	Region Proposal Network
Detectron2-R50	Detectron2 framework using ResNet-50 backbone with FPN	v8n	YOLOv8n-Pose (’v’: version, ’n’: nano)
ED	Euclidean Distance	v11n	YOLOv11n-Pose (’v’: version, ’n’: nano)
EffCSP	Efficient Cross Stage Partial	w/ aug.	With Augmentation
FPN	Feature Pyramid Network	w/o aug.	Without Augmentation
IoU	Intersection over Union	YOLO	You Only Look Once

### Dataset description

This study utilizes the publicly available *Spondylolisthesis Vertebral Landmark* dataset [38], which comprises a total of 716 sagittal (lateral) lumbar spine X-ray images collected from two sources. The first subset includes 208 images from a proprietary dataset of Honduran patients diagnosed with spondylolisthesis. The second subset consists of 508 images from the publicly available BUU-LSPINE dataset, restricted to sagittal views only, as indicated by the dataset description. The dataset includes a demographically diverse set of male and female patients.

Each image was standardized to a resolution of 640 × 640 pixels and manually annotated by the dataset authors. Vertebrae were labeled with bounding boxes and four anatomical corner keypoints per instance. Annotations are provided in JSON format, compatible with PyTorch’s Keypoint R-CNN pipeline.

Out of the 716 total images, 698 were fully annotated and used for model training and evaluation, while the remaining 18 images were reserved for clinical expert review or lacked complete annotations. The dataset is released under a CC BY 4.0 license and is available from the Mendeley Data repository (download size: approximately 41.9 MB).

For model comparison, all 698 fully annotated images were preprocessed to support the effective training of keypoint detection models such as YOLO and Detectron2. In most keypoint detection frameworks, each keypoint is assigned a visibility flag: 0 denotes not visible, 1 partially visible (e.g., due to occlusion or poor contrast), and 2 fully visible. In this dataset, all keypoints were verified to be fully visible.

Given the relatively small dataset size, an 80/10/10 split was adopted for training, validation, and testing, respectively. This configuration maximizes training data while maintaining sufficient samples for reliable validation and unbiased performance evaluation.

**Fig 1:**
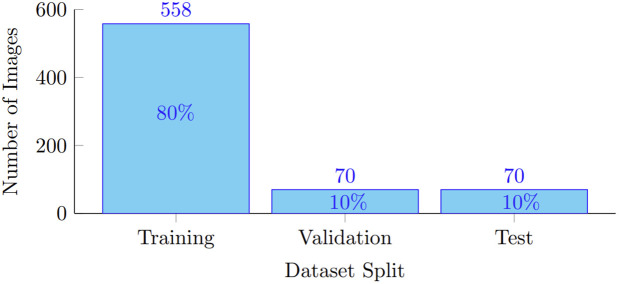
Image distribution across dataset splits: The 698 fully annotated images were divided into training, validation, and test sets.

A single fixed split was intentionally used to ensure identical training and evaluation conditions across all models, enabling a fair and controlled comparative analysis rather than optimizing individual model performance. The distribution of images across the dataset splits is illustrated in Fig 1.

### Annotation format

To ensure compatibility with multiple model architectures, the original annotations (provided in PyTorch Keypoint R-CNN JSON format) were converted into two framework-specific formats using custom deterministic preprocessing scripts. This conversion preserves identical bounding box geometry, keypoint ordering, and visibility semantics, ensuring a one-to-one correspondence across all models. The following formats were generated:

- **YOLO keypoint format:** This format was used for training the YOLOv8n-Pose and YOLOv11n-Pose models. It encodes bounding box centroids, dimensions, and keypoint coordinates *normalized* to the range [0, 1] relative to the image dimensions, along with visibility flags for each keypoint. The annotation line follows the structure:
  <class_id> <x_center> <y_center> <width> <height> <kpt1_x> <kpt1_y> <kpt1_visible > ... < kptN_x> <kptN_y> <kptN_visible > .
   *Example with 4 keypoints per bounding box:*
 1 0.3156 0.6797 0.1219 0.2563 0.3297 0.7031 2 0.3594 0.7063 2 0.3672
 0.7500 2 0.3281 0.7734 2
- **COCO JSON format:** This format was used for Detectron2’s Keypoint R-CNN models with ResNet-50 FPN and ResNet-101 FPN backbones. It encodes bounding boxes and keypoints in absolute pixel coordinates and adheres to the standard COCO annotation schema. Each keypoint is represented as a triplet [x, y, visibility].
   *Example with 4 keypoints per bounding box:*
{
“annotations”: [
{
“image_id”: 1,
“category_id”: 1,
“bbox”: [202, 435, 78, 164],
“keypoints”: [211, 450, 2, 230, 452, 2, 235, 480, 2, 210, 495, 2],
“num_keypoints”: 4
  }
  ]
  }
  This process ensured compatibility with each model’s required input format while maintaining consistent annotations across all models.

### Compared models and architectures

This study benchmarks four pose-based object detection models for vertebrae detection using bounding box and keypoint annotations. The selected models span both single-stage and two-stage detection paradigms and include lightweight as well as deeper architectures commonly used for keypoint detection in medical imaging.

### Overview of models

- **YOLOv8n-Pose:** A lightweight single-stage model from the YOLOv8 family, optimized for real-time keypoint detection with minimal computational cost. In the code, it is referred to as yolov8n-pose, where the “n” indicates the nano variant.
- **YOLOv11n-Pose:** A newer variant of YOLOv8n-Pose with an improved backbone and updated feature fusion and attention modules. In the code, it is referred to as yolo11n-pose, without the letter “v”. The “n” again indicates the nano variant. These architectural updates are designed to improve detection capabilities while maintaining low inference latency.
- **Detectron2 (Keypoint R-CNN with ResNet-50 FPN):** A two-stage keypoint detection model using a ResNet-50 backbone combined with a Feature Pyramid Network (FPN), implemented via the Detectron2 framework.
- **Detectron2 (Keypoint R-CNN with ResNet-101 FPN):** A deeper variant of the previous architecture using a ResNet-101 backbone, which potentially improves feature representation and localization accuracy at the cost of increased computational complexity.

These models were selected to assess trade-offs between detection accuracy, computational efficiency, and robustness in vertebrae keypoint localization. All models were initialized using publicly available pretrained weights provided by their respective frameworks and subsequently fine-tuned on the vertebrae dataset. This transfer learning strategy follows standard practice in medical imaging to mitigate overfitting when training deep architectures on limited datasets.

As summarized in Table 2, YOLO models use a single-stage, anchor-free detection approach with direct coordinate regression, enabling faster inference. In contrast, Detectron2 employs a two-stage anchor-based pipeline using a Region Proposal Network (RPN) and heatmap-based keypoint localization, which may provide more spatially consistent predictions at the cost of slower inference speed. This comparison provides context for the architectural diagrams presented in the next subsection.

**Table 2:** Comparison of anchor-free and anchor-based architectures for vertebrae detection.

Aspect	YOLOv8-Pose/YOLOv11-Pose (Anchor-Free)	Detectron2 (Keypoint R-CNN, Anchor-Based)
Anchor Usage	No anchors	Uses anchors via Region Proposal Network (RPN)
Inference Speed	Faster (single-stage)	Slower (two-stage)
Accuracy	Effective for structured tasks	Strong performance on COCO-style datasets
Training Complexity	Simple, no anchor tuning	Requires tuning of anchor sizes/aspect ratios
Keypoint Prediction	Direct regression of (*x*, *y*)	Heatmap-based localization
Spatial Smoothness	Raw coordinates, less smoothing	Smoother via Gaussian heatmaps
Small Object Handling	Adequate resolution required	Often better due to RoI-alignment
Output Style	Coordinates per keypoint	Heatmaps converted to pixel locations
Object Scale Handling	Learned directly	Managed by FPN + anchors

### Architecture diagrams

To clarify the internal mechanisms of each model, architectural diagrams are provided to illustrate the main detection and keypoint estimation components used in YOLO-based and Detectron2-based frameworks.

**Fig 2:**
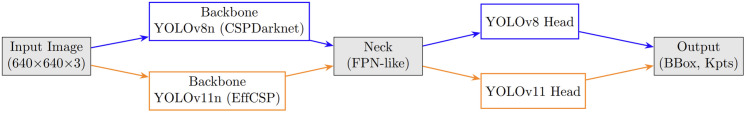
Unified view of YOLOv8n-Pose and YOLOv11n-Pose architectures: Shared components are in gray, YOLOv8n path in blue, and YOLOv11n path in orange.

- **YOLOv8n-Pose and YOLOv11n-Pose:** These are single-stage models where detection and keypoint estimation are performed simultaneously. The overall pipeline includes a backbone for feature extraction, a neck (FPN-like structure) for multi-scale feature fusion, and task-specific heads for joint bounding box and keypoint prediction. The architecture is illustrated in Fig. 2.

**Prediction tensor and training loss in YOLOv8n/YOLOv11n-Pose:**

Each prediction *head* outputs a tensor of shape (S×S×(B·(5+K·3))), where:

- *S* × *S* the grid size of the feature map,
- *B* the number of bounding boxes predicted per grid cell,
- 5 represents the bounding box attributes: (*x*, *y*, width, height, confidence),
- *K* is the number of keypoints per object,
- 3 encodes (*x*, *y*, visibility) for each keypoint.

The total loss ℒ used during training is a weighted sum:


$$ℒ=\lambda_{box}\cdot {ℒ}_{box}+\lambda_{obj}\cdot {ℒ}_{obj}+\lambda_{kpt}\cdot {ℒ}_{kpt}$$

where ${ℒ}_{box}$ is the bounding box loss (CIoU), ${ℒ}_{obj}$ is the objectness loss (BCE), and ${ℒ}_{kpt}$ is the keypoint loss (OKS-weighted Euclidean distance), computed over visible keypoints.

**Fig 3:**
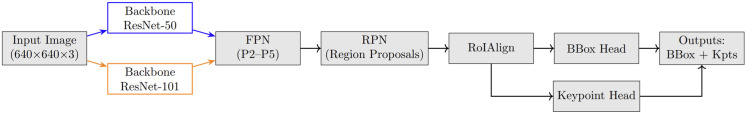
Detectron2 keypoint R-CNN architecture with R-50 and R-101 FPN backbones: Blue indicates the ResNet-50 path, orange indicates the ResNet-101 path, and gray shows shared components.

- **Detectron2 with keypoint R-CNN:** It is a two-stage framework where region proposals are generated using an RPN, followed by refined detection and keypoint estimation. The backbone can be either ResNet-50 or ResNet-101, both enhanced with an FPN. The full pipeline is shown in Fig 3.

### Prediction tensor and training loss in Detectron2 keypoint R-CNN:

The keypoint head outputs a tensor of shape


N×K×H×W,

where *N* is the number of region proposals, *K* is the number of keypoints, and *H* × *W* is the spatial resolution of the predicted *heatmap* for each keypoint. Final keypoint coordinates are obtained by selecting the maximum response in each *heatmap*.

The overall loss is defined as:


$$ℒ={ℒ}_{cls}+{ℒ}_{bbox}+{ℒ}_{kpt}$$

- ${ℒ}_{cls}$: classification loss (cross-entropy),

- ${ℒ}_{bbox}$: bounding box regression loss (Smooth L1),

- ${ℒ}_{kpt}$: keypoint loss, computed as:


$${ℒ}_{kpt}=\frac{1}{K}\sum_{k=1}^{K}1(v_{k}\geq 1)\cdot {\left\|{\hat{𝐩}}_{k}-{𝐩}_{k}\right\|}_{1}$$

with $v_{k}\in \{0,1,2\}$ as the visibility flag for keypoint *k*, and ${\hat{𝐩}}_{k}$, **p**_*k*_ are the predicted and ground-truth coordinates, respectively. As our dataset contains only fully visible keypoints (*v*_*k*_ = 2), the loss is applied exclusively to these keypoints, while retaining the general COCO-compatible formulation.

### Training details

To rigorously compare the models for vertebrae detection and keypoint estimation, we employed a standardized experimental setup using both original and augmented datasets to assess performance and robustness.

### Experimental environment and standardization

All default data augmentation operations within the YOLO training pipeline were disabled to ensure consistency with the Detectron2 setup and maintain comparable input distributions across frameworks. All models were trained and evaluated under identical experimental conditions, and the software and hardware environment used for training and evaluation is summarized in Table 3.

**Table 3:** Software and hardware setup used for all training and evaluation experiments, ensuring reproducibility.

Software / Library	Version / Notes	Hardware / Env.	Specification
Python	3.8.19	CPU	Intel Core i9 (13th Gen) @ 2.2 GHz
PyTorch	2.0.1	CPU cores / threads	24 cores / 32 threads
CUDA Toolkit	11.7	RAM	32 GB DDR5, 5600 MT/s
cuDNN	8.5.0	OS	Windows 11 Home
Ultralytics YOLO	v8.3.159 (used for YOLOv8n-Pose and YOLOv11n-Pose)	GPU (memory)	NVIDIA GeForce RTX 4060 Laptop GPU (8 GB)
Detectron2	v0.6 (Keypoint R-CNN with ResNet-50/101 backbones)	Storage	Non-Volatile Memory Express (NVMe) SSD, 1 TB

**Note:** Other standard packages (NumPy 1.24.4, OpenCV 4.11.0, etc.) were used as needed.

### Hyperparameter alignment

We carefully aligned training parameters across the YOLO and Detectron2 frameworks, including batch size, optimizer, input resolution, and learning rate schedules, as summarized in Table 4. Wherever possible, default settings were retained to minimize tuning bias and preserve the inherent behavior of each framework. While the core training pipelines were based on recommended practices, minor task-specific adaptations were introduced to better accommodate anatomical keypoint detection.

**Table 4:** Detailed training configurations for pose estimation models. This table corresponds to the results presented in Table 6.

Parameter	YOLOv8n-Pose / YOLOv11n-Pose	Detectron2 (Keypoint R-CNN, ResNet-50 / ResNet-101 FPN)
Framework	Ultralytics YOLOv8	Detectron2
Pretrained Weights Source	COCO-pretrained weights (YOLOv8 model zoo)	COCO-pretrained weights (Detectron2 model zoo)
Annotation Format	Normalized YOLO keypoints	COCO format (absolute pixel coordinates)
Parameter (Trainable / Total), in millions (M)	v8n: 3.30M/3.30M; v11n: 2.87M/2.87M	R50: 58.9M/59.1M; R101: 77.9M/78.1M
Input Resolution	640 × 640	640 × 640
Batch Size	8	8
Learning Rate	0.01 (default)	0.01
Anchor Sizes	Not used	[[32], [64], [128], [256], [512]] (default)
Anchor Ratios	Anchor-free architecture (no explicit anchors used)	[0.5, 1.0, 2.0] (default)
Data Loader Workers	8 (default, if supported by system)	8
Keypoint OKS Tolerance (σ)	YOLO default, Not exposed	[0.05, 0.05, 0.05, 0.05] (equivalent)
Loss Function	Composite loss: objectness, box regression, keypoints (built-in)	Keypoint R-CNN losses: classification, box, keypoint (built-in)
Keypoint Weight	Automatically balanced in YOLO loss	Fixed at 1.0 (default)
Optimizer	SGD (default)	SGD (default)
LR Scheduler	Cosine annealing with warmup (default)	WarmupCosineLR (aligned with YOLO-Pose via custom set)
AMP (mixed precision)	Enabled (default)	Enabled
Note: The number of iterations per epoch in Detectron2 is computed as $\lceil \frac{\text{Number of Training Images}}{\text{Batch Size}}\rceil =\lceil \frac{558}{8}\rceil =70$. Thus, 1 epoch ≈ 70 iterations.		
Epochs (max)	100	100 (MAX_ITER = 70 × 100 = S
Evaluation Frequency	Every 1 epoch (default)	Every 2 epochs (70 × 2 = 140 iters)
Early Stopping Metric	keypoint mAP@0.5:0.95 (default)	keypoint mAP@0.5:0.95 (custom-set)
Early Stopping	Enabled (patience = 20 epochs)	Enabled (patience = 20 epochs) via custom hook (i.e., 140 × 10 = 1400 iters)
Warmup Factor	Automatically set in YOLO	0.001 (quite close to YOLOv8)
Warmup Iteration	3 (default)	3 (WARMUP_ITERS=3×70=210 iters)
Warmup Method	Linear (default, not exposed)	Linear

**Note:** All experiments used a global random seed of 42, unless explicitly mentioned otherwise, to ensure consistent initialization and fair comparison across models.

### Data augmentation and dataset expansion

To enhance generalization, a focused set of data augmentation techniques was applied as a preprocessing step prior to dataset conversion. These included random rotation, horizontal flipping, and brightness adjustments (see Table 5). The augmented data was then converted into the respective input formats for YOLO and Detectron2 to ensure that both frameworks were trained on an identical augmented dataset. These clinically appropriate augmentations were selected to improve model robustness against typical variations in patient positioning and X-ray imaging conditions.

**Table 5:** Training configuration under data augmentation. Remaining parameters are as in Table 4. This table corresponds to the results presented in Table 7.

Parameter	YOLOv8n-Pose / YOLOv11n-Pose	Detectron2 (Keypoint R-CNN, ResNet-50 / ResNet-101 FPN)
Augmentation Type for *all models* (3 augmentations per image; probability (p))	• Random Rotate 90° (p = 1.0)	
	• Horizontal Flip (p = 0.5)	
	• Random Brightness/Contrast (±30%, p = 1.0)	
Epochs (max)	100	100 (MAX_ITER = 100 279 = 27900)
Evaluation Frequency	Every 1 epoch (default)	Every 2 epochs (2 × 279 = 588 iters)
Warmup Iteration	3 (default)	3 (WARMUP_ITERS=3×279=837 iters)
Early Stopping	Enabled (patience = 20 epochs)	Enabled (patience = 20 epochs) via custom hook (i.e., 558 × 10 = 5580 iters)

**Note:** Consistent with Table 4, a global random seed of 42 was applied. The fourfold augmented dataset (2232 images) was preprocessed once, saved, and reused across all models. In this setup, 1 epoch corresponds to 279 iterations.

As part of the experiments detailed in Table 5, the training dataset was augmented fourfold, increasing the number of training images from 558 to 2232 (i.e., 558 × 4), while keeping validation and test sets unchanged to ensure consistent evaluation. This expansion significantly enhanced data diversity, which may lead to improved model stability and faster convergence.

### Training Execution and Monitoring

Early stopping patience was uniformly set to 20 epochs in all experiments, providing a balanced trade-off between computational efficiency and stability. This setting allowed sufficient tolerance for fluctuations in validation loss while preventing premature termination. All models were trained until convergence, and total training time was recorded to assess computational efficiency.

To maintain a consistent evaluation strategy within Detectron2 and reduce computational overhead, we initially set Detectron2 to evaluate every 70 iterations (roughly one epoch), similar to YOLO’s default evaluation frequency. This was considered too frequent and imposed an unnecessary computational burden. After preliminary testing, we adjusted the evaluation interval to every 140 iterations (2 epochs) to reduce overhead while still providing reliable monitoring. The same frequency was applied when training with the augmented dataset to ensure consistency.

The effectiveness of this strategy is demonstrated in the improved quantitative and qualitative results, as detailed in Section Results, highlighting enhanced model robustness and efficiency.

### Detectron2 optimization experiments

In addition to these standardized configurations (see Tables 4 and 5), we conducted further experiments with Detectron2 using optimized hyperparameters. Through detailed observation of convergence patterns, we found that Detectron2 models typically stabilized well before 10 epochs. Therefore, early stopping patience was reduced to 10 epochs for these optimized configurations, effectively lowering training time while preserving accuracy. These results are reported separately in Section Detectron2 keypoint R-CNN (optimized configurations) (see Table 9).

## Results

We evaluated these models through both quantitative metrics and qualitative visualizations to provide a comprehensive assessment of their performance in vertebrae keypoint detection. The results highlight how each approach handles the spatial and anatomical challenges inherent in medical imaging tasks. Visual inspections are included to qualitatively assess predictions in representative clinical scenarios, revealing patterns of success and failure not fully captured by metrics.

In addition, we analyze training dynamics to evaluate model convergence and generalization across architectures. We systematically examine the effects of architectural depth on localization accuracy and robustness, providing an ablation-style analysis of the factors that most influence performance.

### Threshold selection and evaluation

During evaluation and visualization, confidence thresholds were carefully chosen based on empirical observations and applied only to bounding box predictions. For YOLOv8n-Pose and YOLOv11n-Pose, a threshold of 0.5 was used, while for Detectron2 Keypoint R-CNN, a higher threshold of 0.7 was applied to reduce false positives and ensure reliable anatomical keypoint detection. Since each bounding box contains exactly four keypoints, keypoint predictions are automatically affected by the box threshold. For Detectron2, each keypoint also has a confidence score, so it would be possible to apply an additional keypoint-specific threshold if stricter filtering were desired.

### Quantitative evaluation

To provide a thorough evaluation of vertebrae localization and keypoint estimation, we employ a comprehensive set of evaluation metrics that combine both COCO-style pose estimation standards and custom criteria tailored for medical imaging.

### Evaluation metrics and justification

Given the dual task of vertebra detection and anatomical keypoint localization, we report:

- **Bounding box metrics:** Mean Average Precision (mAP) at IoU thresholds of 0.5:0.95 and 0.5, along with precision, recall, and F1-score computed at IoU ≥ 0.5. These metrics allow standardized comparison and help distinguish false positives from false negatives.
- **Keypoint metrics:** OKS-based mAP@0.5 and mAP@0.5:0.95 are used to assess fine-grained localization accuracy. In addition, we report keypoint-level precision, recall, and F1-score (using OKS ≥ 0.5), and accuracy based on Euclidean distance thresholds (5 px, 10 px, and 15 px) to offer clinical interpretability.

Notably, prior studies often omit or loosely define the spatial thresholds used for keypoint accuracy, which can lead to misleadingly high performance claims under lenient conditions. To address this, we adopt clearly defined and progressively stricter thresholds.

These distance thresholds reflect varying levels of tolerance for anatomical localization:

- **5 pixels:** Strict precision, suitable for high-confidence clinical measurements.
- **10 pixels:** Moderate tolerance, balancing precision with flexibility.
- **15 pixels:** Lenient threshold, accounting for inter-observer variability and imaging noise.

This stratification allows for a more comprehensive and realistic assessment of keypoint accuracy in medical settings.

- **Inference and training efficiency:** Average inference time per image (in milliseconds), best epoch, and total training time per model illustrate suitability for real-time applications and highlight differences in computational efficiency across frameworks.

### Sequential evaluation procedure

To reduce noise and improve interpretability, we adopt a sequential evaluation strategy. Bounding box matches (IoU ≥ 0.5) are first identified; only keypoints within these matched boxes are then considered for evaluation. This avoids penalizing keypoints predicted in irrelevant or incorrect locations and better reflects real-world diagnostic utility. This sequential filtering is particularly beneficial in medical imaging tasks, where accurate object localization must precede reliable anatomical landmark detection.

Furthermore, to ensure an unbiased and consistent comparison between YOLO and Detectron2, we did not rely on the validation metrics reported directly during YOLO’s training phase. Instead, predictions from both models were exported in COCO JSON format and re-evaluated using the official COCO evaluation API under identical conditions. This procedure eliminates framework-specific differences in metric computation and guarantees that reported values (e.g., mAP@0.5) are directly comparable across models.

### Dataset splits

Metrics are reported separately for validation and test sets. Validation reports only mAP metrics for model selection, while the test set reports the full metric suite for final evaluation. This separation is essential in clinical contexts, where data scarcity and inter-patient variability make generalization a critical concern. The results on the original and augmented datasets are summarized in Tables 6 and 7, respectively.

**Table 6:** Quantitative evaluation of models on the original dataset. Metrics are reported separately for validation and test sets to assess both model performance and generalization. Bounding box and keypoint results are expressed as percentages (%), while inference time, best epoch, and training time provide insights into computational efficiency.

Set	Metric	YOLOv8n-Pose	YOLOv11n-Pose	Detectron2-R50	Detectron2-R101
Validation	**Bounding Box Metrics**				
	mAP@0.5:0.95	**63.30**	60.40	56.30	57.20
	mAP@0.5	92.10	91.80	**92.20**	91.60
	**Keypoint Metrics**				
	mAP@0.5:0.95 (OKS)	**69.30**	66.50	33.80	36.70
	mAP@0.5 (OKS)	**72.00**	**72.00**	55.60	59.30
Test	**Bounding Box Metrics**				
	mAP@0.5:0.95	59.70	**60.80**	56.50	59.20
	mAP@0.5	90.00	91.90	91.40	**93.00**
	Precision (IoU ≥ 0.5)	91.06	**92.29**	82.79	87.83
	Recall (IoU ≥ 0.5)	87.83	87.39	94.69	**95.80**
	F1-score (IoU ≥ 0.5)	89.41	89.77	88.34	**91.64**
	**Keypoint Metrics**				
	mAP@0.5:0.95 (OKS)	61.40	**63.50**	34.90	32.30
	mAP@0.5 (OKS)	64.70	**67.90**	54.50	51.80
	Precision (OKS ≥ 0.5)	57.80	53.50	**60.93**	60.04
	Recall (OKS≥ 0.5)	55.75	50.66	**69.69**	65.49
	F1-score (OKS ≥ 0.5)	56.76	52.05	**65.02**	62.65
	Accuracy (ED ≤ 5 px)	75.63	**76.75**	75.77	75.06
	Accuracy (ED ≤ 10 px)	98.51	97.37	98.34	98.78
	Accuracy (ED ≤ 15 px)	100.00	**100.00**	**99.78**	99.78
Average Inference Time (ms)		**9.35**	10.43	77.52	91.14
Best Epoch		39	20	08 (560 iters)	64 (4480 iters)
Training Time in Hours (h)		59 epochs: 0.127 h	40 epochs: **0.083 h**	28 epochs (1960 iters): 3.820 h	84 epochs (5880 iters): 9.018 h

**Note:** All keypoint-based precision, recall, F1-score, and accuracy metrics are computed using a custom evaluation approach, which only considers keypoints associated with correctly matched bounding boxes (IoU ≥ 0.5). This strategy aligns with clinical priorities, where anatomical landmarks are only meaningful when spatial localization is accurate. Bold values indicate the top performers.

**Table 7:** Quantitative evaluation of models trained on the augmented dataset. Results illustrate the impact of increased data diversity and can be directly compared with Table 6.

Set	Metric	YOLOv8n-Pose	YOLOv11n-Pose	Detectron2-R50	Detectron2-R101
Validation	**Bounding Box Metrics**				
	mAP@0.5:0.95	59.70	**63.10**	56.50	54.60
	mAP@0.5	90.70	91.40	**92.80**	90.10
	**Keypoint Metrics**				
	mAP@0.5:0.95 (OKS)	60.40	**67.20**	27.10	27.30
	mAP@0.5 (OKS)	63.40	**70.70**	48.50	46.40
Test	**Bounding Box Metrics**				
	mAP@0.5:0.95	**60.10**	59.30	56.80	57.50
	mAP@0.5	89.00	89.10	**93.10**	91.50
	Precision (IoU ≥ 0.5)	**90.79**	89.85	77.26	86.84
	Recall (IoU ≥ 0.5)	89.38	92.04	**96.24**	94.91
	F1-score (IoU ≥ 0.5)	90.08	**90.93**	85.71	90.70
	**Keypoint Metrics**				
	mAP@0.5:0.95 (OKS)	59.90	**60.10**	26.50	30.40
	mAP@0.5 (OKS)	62.00	**62.90**	45.20	47.60
	Precision (OKS ≥ 0.5)	**59.78**	57.45	52.40	59.31
	Recall (OKS≥ 0.5)	58.85	58.85	**65.27**	64.82
	F1-score (OKS ≥ 0.5)	59.31	58.14	58.13	**61.95**
	Accuracy (ED ≤ 5 px)	**79.38**	77.71	73.62	71.74
	Accuracy (ED ≤ 10 px)	96.85	98.17	98.23	**98.45**
	Accuracy (ED ≤ 15 px)	**100.00**	**100.00**	99.83	99.83
Average Inference Time (ms)		**9.45**	12.70	129.88	160.83
Best Epoch		51	62	04 (1116 iters)	26 (7254 iters)
Training Time in Hours (h)		71 epochs: **0.571 h**	82 epochs: 0.636 h	24 epochs (6696 iters): 13.929 h	46 epochs (12,834 iters): 32.351 h

**Note:** Metrics are computed using the same custom evaluation approach as in Table 6. Bold values indicate top performers.

### Performance on original dataset (baseline comparison)

To objectively benchmark the capabilities of each model architecture, we begin by analyzing their performance on the unaltered original dataset. This provides a reliable reference point for understanding model behavior without the influence of data augmentation or synthetic variations. Table 6 outlines the comparative performance metrics of each model on the original dataset, providing a multi-dimensional assessment of model behavior on validation and test sets.

On the validation set, both YOLOv8n-Pose and YOLOv11n-Pose achieved the highest overall mAP@0.5 for keypoint estimation (72.00%), while YOLOv8n-Pose also reached 92.10% for bounding box detection, indicating strong spatial and anatomical accuracy. Interestingly, Detectron2-R50 yielded the best mAP@0.5 for bounding boxes (92.20%), slightly outperforming both YOLO variants at that threshold.

Test set results show a different pattern: YOLOv11n-Pose outperformed others in mAP@0.5:0.95 for both bounding boxes (60.80%) and keypoints (63.50%), as well as in bounding box precision (92.29%). However, Detectron2-R101 reported the highest recall (95.80%) and F1-score (91.64%) for bounding box detection, indicating strong object-level coverage. For keypoints, Detectron2-R50 achieved the best F1-score (65.02%) and recall (69.69%), suggesting improved anatomical completeness.

Keypoint accuracy measured via Euclidean distance reveals minimal differences at high thresholds: all models achieved nearly 100% accuracy at a 15-pixel threshold, while YOLOv11n-Pose slightly led at 5 pixels (76.75%).

From a computational perspective, YOLOv11n-Pose was by far the most efficient, with an average inference time of just 10.43 ms per image, at least 7 × faster than the Detectron2 models and nearly the same as YOLOv8n-Pose.

Together, these results establish YOLOv11n-Pose as a strong balance between accuracy and efficiency on the original dataset, while Detectron2 models offer high recall and keypoint F1-scores, which may be preferable in clinical contexts requiring exhaustive anatomical localization.

### Performance on augmented dataset (impact of light augmentation)

Building on the baseline results, this section explores the impact of data augmentation on model performance. Augmentation was introduced to increase anatomical variability, enhance generalization, and mitigate overfitting, which is particularly important in limited medical datasets. Table 7 presents the results of all four models after training on the augmented dataset. These can be directly compared with Table 6 to assess the effects of increased data diversity.

On the validation dataset, YOLOv11n-Pose showed mixed results with augmentation: bounding box mAP@0.5 improved from 60.40 to 63.10, while keypoint mAP@0.5 (OKS) decreased slightly from 72.00 to 70.70. YOLOv8n-Pose declined in both metrics (mAP@0.5 from 92.10 to 90.70; mAP@0.5 (OKS) from 72.00 to 63.40). For Detectron2-R50, augmentation had minimal effect on bounding box mAP@0.5 (92.20 to 92.80) but caused a decline in keypoint mAP@0.5 (OKS) from 55.60 to 48.50. Detectron2-R101 also showed reduced performance across both metrics (mAP@0.5 from 91.60 to 90.10; mAP@0.5 (OKS) from 59.30 to 46.40). A similar trend was observed on the test dataset, indicating that even light augmentations such as random 90° rotation, horizontal flip, and brightness/contrast adjustments can perturb keypoint localization, particularly for models more sensitive to spatial transformations.

**Fig 4:**
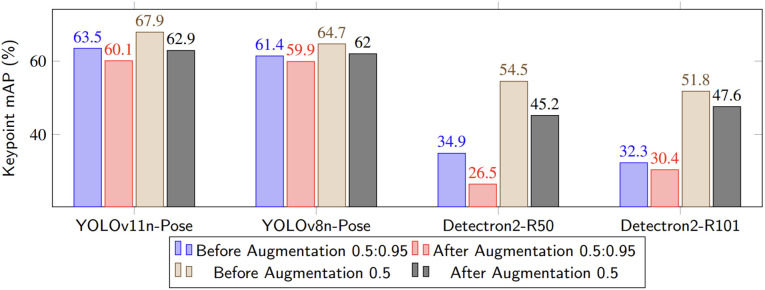
Keypoint mAP before and after augmentation: Results are shown for both strict (0.5:0.95) and loose (0.5) thresholds on the test set.

Overall, augmentation resulted in *noticeable declines in keypoint localization accuracy across all models*. In contrast, bounding box performance was mostly stable, with slight increases in some cases and minor decreases in others, indicating that the augmentations primarily introduced spatial noise affecting fine-grained anatomical feature detection (see Fig 4).

In terms of inference speed, both YOLOv8n-Pose and YOLOv11n-Pose maintained real-time performance with inference times below 15 ms, whereas Detectron2 models were much slower, taking at least 10 times longer per image. Augmentation had only a slight effect on inference times for YOLO models and a moderate effect on Detectron2 models.

Taken together, these findings highlight the mixed impact of augmentation: it improves bounding box generalization in some cases but shows limited or inconsistent benefits in others, while reducing keypoint precision. Among the evaluated models, **YOLOv11n-Pose** achieved the best overall balance post-augmentation, maintaining strong bounding box performance and comparatively stable keypoint accuracy. Nonetheless, even light augmentation decreased keypoint localization across all models, with stronger augmentation conditions likely to amplify this effect, as discussed in Section Discussion.

### Comparative analysis of models

A direct comparison of the evaluated architectures reveals substantial differences in computational efficiency, with YOLO-based models demonstrating markedly faster training times than Detectron2 variants (see Tables 6 and 7).

YOLO models (v8n and v11n) reached convergence in under 40 minutes, even with the augmented (larger) dataset, whereas Detectron2 models required significantly longer, ranging from approximately 4–33 hours. This stark difference highlights the superior computational efficiency of YOLO-based architectures under standardized training conditions.

Despite high recall in bounding boxes, Detectron2 models show comparatively lower effectiveness in keypoint localization, particularly under OKS-based evaluation. This may be attributed to architectural characteristics or pretraining on datasets not optimized for vertebral landmarks.

All models were trained and evaluated under carefully aligned settings (see Tables 4 and 5) to ensure consistent comparisons. While alternative strategies might yield different outcomes for larger models such as ResNet-101, YOLOv11n-Pose remains the most effective under the standardized configurations tested.

Further analysis, including ablation-type evaluations of hyperparameters and training duration, reinforces these findings (see Section Comparison of model variants).

## Qualitative results

In addition to the quantitative evaluation, we provide a qualitative assessment of the models’ performance to visually illustrate their effectiveness in vertebrae detection and anatomical keypoint localization.

The visualizations include bounding boxes around detected vertebrae and keypoints marked on relevant anatomical landmarks. Qualitative analysis highlights the models’ ability to accurately localize vertebrae under varying image conditions, as well as their precision in identifying all keypoints consistently across images.

**Fig 5:**
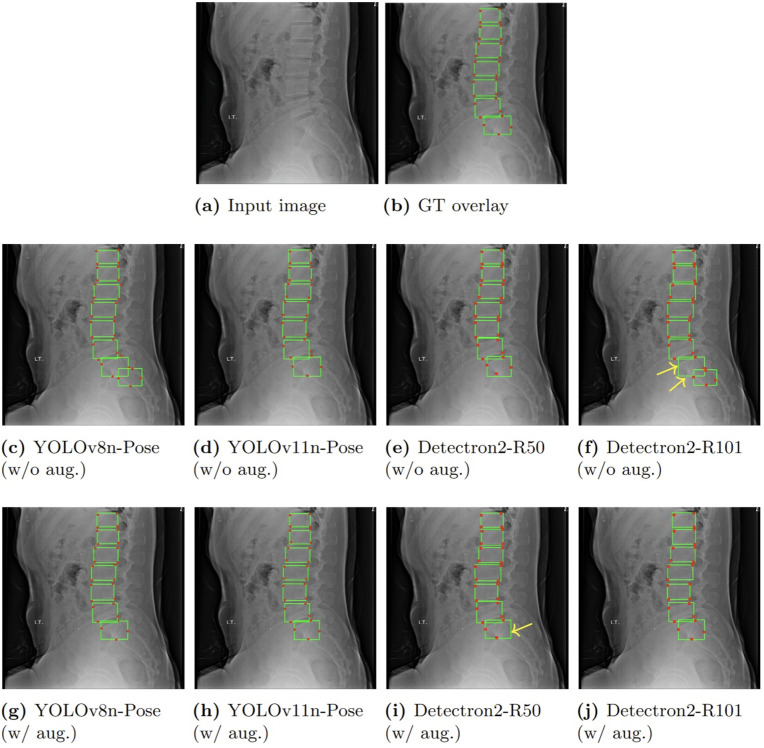
Qualitative comparison of model predictions in a simple case with and without augmentation: The top row shows the input image and ground truth (GT) overlay. The middle row shows predictions from models trained without augmentation, while the bottom row shows results with augmentation. Predicted bounding boxes are in green, with red dots indicating keypoints. Predictions are visualized using an IoU threshold of 0.5 for bounding boxes and an OKS threshold of 0.5 for keypoints. All models perform well here; however, YOLOv8n-Pose and Detectron2-R101 without augmentation show one extra bounding box with keypoints compared to the ground truth. After augmentation, all bounding boxes and keypoints align correctly. Yellow arrows highlight keypoints that appear missing, usually due to occlusion rather than prediction failure.

**Fig 6:**
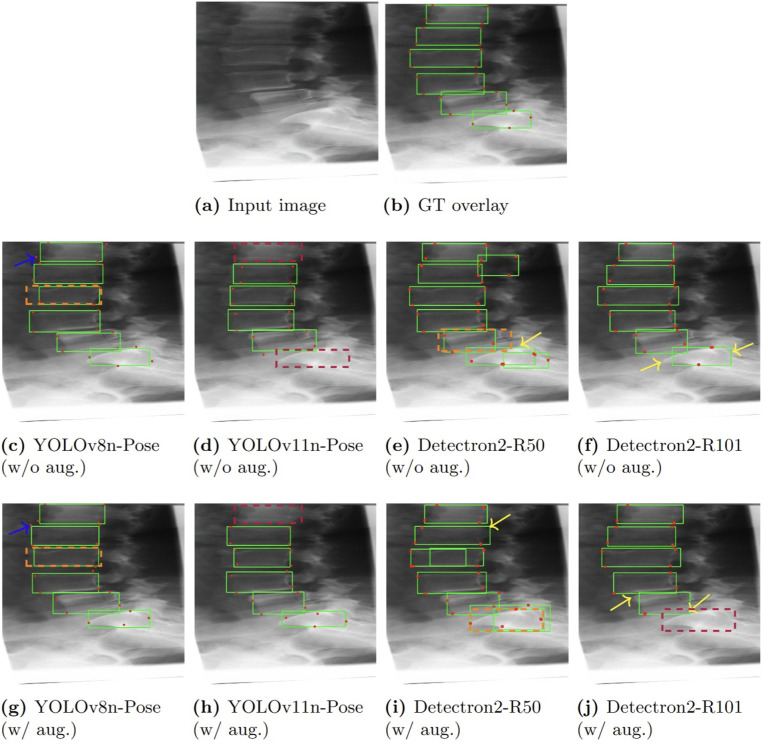
Qualitative results for a challenging case: Performance of different models with and without augmentation in vertebrae detection and keypoint localization(row layout as in Fig 5). Predictions are visualized using an IoU threshold of 0.5 for bounding box matching and an OKS threshold of 0.5 for keypoint evaluation. Blue arrows highlight incorrectly predicted keypoints. Yellow arrows indicate keypoints that appear missing, usually due to occlusion rather than prediction failure. Orange dotted boxes mark expected vertebra locations, and purple boxes denote missing detections. In cases with missing detections, only purple outlines are shown without keypoints to reduce visual clutter. Only a subset of representative errors is annotated for clarity.

To assess performance across a range of conditions, we present two example predictions. Fig 5 depicts a *simple case* where all models perform well, both with and without data augmentation. In contrast, Fig 6 shows a *challenging case* with reduced lighting and anatomical overlap, where models vary more distinctly in prediction quality.

Predicted bounding boxes are shown in green, and red markers denote keypoints. Blue arrows indicate keypoints with significant positional error, while yellow arrows highlight keypoints that appear missing, often due to occlusion rather than an actual failure in prediction. Orange dotted boxes represent the expected ground-truth bounding box locations, and purple boxes indicate missed detections. Each model predicts four keypoints per bounding box, but due to overlap or occlusion, not all may be visually distinct. To avoid clutter, only a few representative errors are annotated per image.

### Observed trends in keypoint prediction

A noteworthy trend across both figures is that YOLO-based models consistently predict all four keypoints per detection (with minimal overlap), demonstrating strong keypoint completeness. However, these models occasionally exhibit minor positional inaccuracies (highlighted by blue arrows). Conversely, Detectron2 models tend to produce more spatially accurate keypoints but occasionally predict fewer than four keypoints for a given vertebra, mainly due to occlusions in the images (as indicated by yellow arrows).

Among all evaluated models, **YOLOv11n-Pose stands out** for its consistent and accurate performance in both bounding box detection and keypoint localization. It balances detection reliability with fine-grained anatomical precision, even under complex imaging conditions involving occlusion and low contrast. This robustness is observed consistently across multiple test cases, reinforcing the model’s suitability for vertebrae localization in real-world clinical imaging applications. Notably, YOLOv8n-Pose achieves comparable results in many cases, making it a strong secondary option when faster inference is prioritized.

These qualitative findings closely align with the quantitative results presented earlier and further underscore YOLOv11n-Pose’s practical advantages for applications that demand both high detection accuracy and precise keypoint estimation.

### Impact of augmentation

Consistent with our quantitative analysis, the qualitative results also confirm that augmentation did not provide a clear positive impact in this task, often introducing spatial variability that reduced keypoint precision, as observed in the comparative rows of Fig 5 and Fig 6.

### Loss convergence analysis

**Fig 7:**
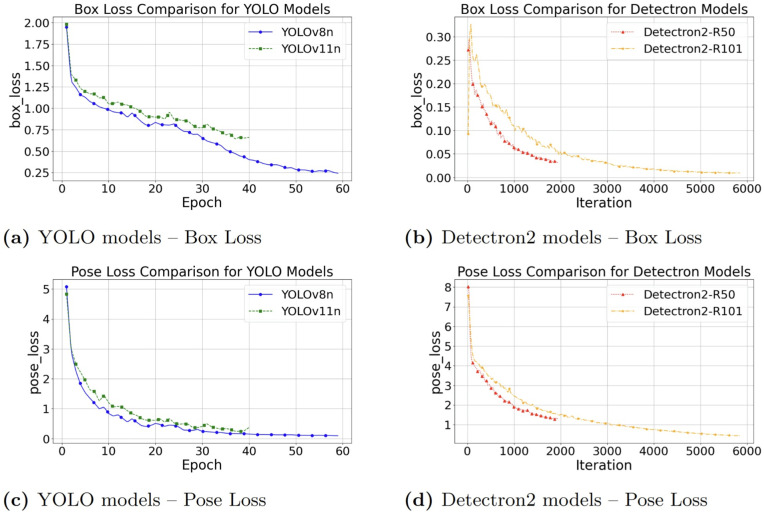
Comparison of box loss and pose loss on the original dataset: Loss curves for YOLO (v8n, v11n) and Detectron2 (R50, R101) models are shown, illustrating convergence behavior during training.

**Fig 8:**
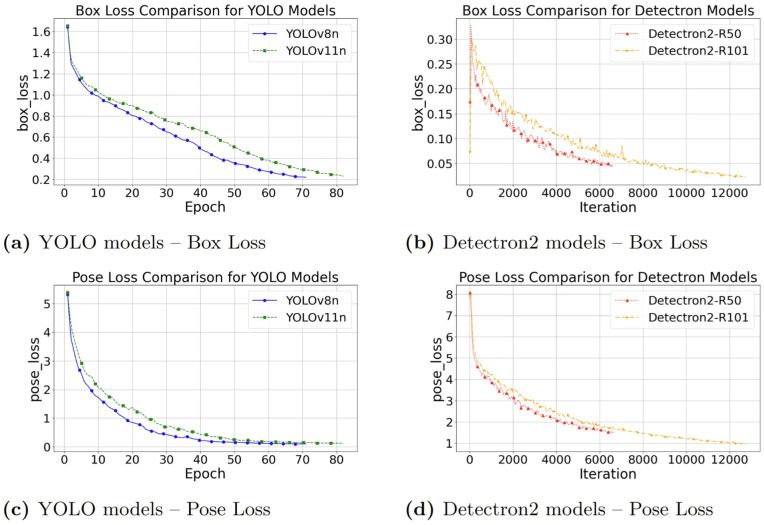
Comparison of box loss and pose loss on the augmented dataset: Loss curves for YOLO (v8n, v11n) and Detectron2 (R50, R101) models are shown, illustrating the effect of data augmentation on training convergence.

Fig 7 illustrates the convergence of box and pose (keypoint) losses for YOLO (v8n, v11n) and Detectron2 (R50, R101) models trained on the original dataset, while Fig 8 shows the corresponding results for models trained on the augmented dataset.

On the original dataset (Fig 7), YOLO models exhibited faster initial convergence in both box and pose losses. Detectron2 models, particularly R101, showed slower but more stable box loss reduction.

With the introduction of data augmentation (Fig 8), both architectures exhibited smoother and more stable convergence profiles. Augmentation reduced oscillations and improved generalization, with modest improvements observed particularly for YOLOv11n-Pose.

Across both the original and augmented datasets, the final loss values remained within consistent ranges: YOLO stabilized between 0.25–0.65 for box loss and 0.25–0.40 for pose loss, whereas Detectron2 achieved substantially lower box losses (0.025–0.045) but comparatively higher pose losses (0.70–1.25).

Overall, data augmentation did not dramatically reduce the final range of losses but helped smooth the loss curves and stabilize training.

**Overall, four key trends are observed:**

- **Rapid convergence of YOLO architectures:** Competitive pose loss is reached in fewer epochs compared to Detectron2-based models.
- **Effect of augmentation on Detectron2:** Bounding box stability is slightly improved with data augmentation.
- **Task-specific trade-off:** Slightly better bounding box prediction is achieved by Detectron2, while YOLO excels overall in both bounding box regression and keypoints.
- **Training time considerations:** Increased training time is required when using data augmentation due to more epochs needed for convergence.

Considering these trends, YOLOv11n emerges as the most effective model overall, offering a strong balance between fast convergence, low pose loss, and good generalization.

### Comparison of model variants

To provide a comprehensive evaluation of vertebrae detection using pose-based models, we compared multiple YOLO pose variants with the optimized configurations of Detectron2 Keypoint R-CNN (using ResNet-50 and ResNet-101). Here, we focus on the strongest settings identified for each framework. This section reports performance on the original test set and highlights differences in accuracy, computational efficiency, and training dynamics.

### YOLO pose estimation variants

We assessed several additional variants beyond the baseline models, including the following: YOLOv8-Small (YOLOv8s), YOLOv8-Medium (YOLOv8m), YOLOv8-Large (YOLOv8l), YOLOv8-Extra Large (YOLOv8x), YOLOv11-Small (YOLOv11s), YOLOv11-Medium (YOLOv11m), YOLOv11-Large (YOLOv11l), and YOLOv11-Extra Large (YOLOv11x). These models were trained under the same settings described in Table 4.

Performance metrics include mean Average Precision (mAP) at an IoU threshold of 0.5 (mAP@0.5) and across multiple thresholds (mAP@0.5:0.95) for both bounding boxes and keypoints, along with total training time measured up to the final epoch selected by early stopping (patience = 20). To facilitate a rapid comparison across multiple model variants, all values are extracted directly from the training logs rather than the official COCO-style evaluation API. For completeness, the baseline nano variants (YOLOv8n and YOLOv11n) are also included, which show slight changes compared to the values reported in Tables 6 and 7. This approach provides an overview of performance across variants while maintaining brevity.

The results, summarized in Table 8, highlight key trade-offs between model size, accuracy, and computational efficiency. Similar trends were observed when training on the augmented dataset, indicating robustness across data variations; however, these details are omitted here for brevity.

**Table 8:** Performance of additional YOLO variants on the validation set (original dataset). All parameters are consistent with those in Table 4.

Model	mAP@0.5:0.95 (BBox)	mAP@0.5 (BBox)	mAP@0.5:0.95 (Keypoints)	mAP@0.5 (Keypoints)	Total Training Time in Hours (h)
YOLOv8n-Pose	62.80	93.40	68.70	71.80	59 epochs: **0.127 h**
YOLOv8s-Pose	60.60	93.40	65.80	69.50	52 epochs: 0.216 h
YOLOv8m-Pose	62.70	93.10	69.50	73.10	42 epochs: 0.421 h
YOLOv8l-Pose	**65.40**	**94.70**	**78.80**	**81.90**	46 epochs: 0.680 h
YOLOv8x-Pose	62.60	92.70	70.40	74.90	35 epochs: 0.961 h
YOLOv11n-Pose	58.70	**93.90**	65.30	73.00	40 epochs: **0.083 h**
YOLOv11s-Pose	**63.20**	92.20	68.30	71.00	85 epochs: 0.309 h
YOLOv11m-Pose	61.20	92.20	67.10	70.10	45 epochs: 0.432 h
YOLOv11l-Pose	62.60	92.20	**71.90**	**75.30**	72 epochs: 0.815 h
YOLOv11x-Pose	60.70	92.00	70.00	73.30	50 epochs: 8.254 h

**Note:** Metrics are reported directly from training logs. Bold values indicate the top performers within each category for each YOLO version (v8 and v11) separately.

### Overall observations of YOLO families

- **YOLOv8 family excels in keypoint detection:** YOLOv8l achieves the highest scores among all variants, with mAP@0.5 of 94.70 for bounding boxes and 81.90 for keypoints, and mAP@0.5:0.95 of 65.40 (BBox) and 78.80 (Keypoints). It outperforms all other models in fine-grained anatomical localization.
- **Other YOLOv8 variants:** YOLOv8m and YOLOv8x maintain competitive object detection performance but have slightly lower bounding box accuracy and relatively poorer keypoint results.
- **YOLOv11 variants:** These show more consistent but slightly lower bounding box accuracy (92.00–93.90 mAP@0.5). YOLOv11l leads in keypoint performance, with mAP@0.5 = 75.30 and mAP@0.5:0.95 = 71.90.
- **Nano variants (YOLOv8n, YOLOv11n):** These models achieve competitive bounding box detection (mAP@0.5 of 93.40 and 93.90, respectively) while requiring minimal training time, making them highly suitable for resource-constrained deployments.
- **Training efficiency scales with model size:** Nano variants converge fastest, YOLOv11n in just 0.083 hours over 40 epochs, followed by YOLOv8n at 0.127 hours. As model size increases, training times rise significantly, peaking at 8.254 hours for YOLOv11x over 50 epochs, reflecting the increased parameter count and computational complexity.
- **Extra-large variants are not guaranteed to perform best:** Despite their size, YOLOv8x and YOLOv11x do not always improve accuracy, while incurring substantially higher training overhead.

Selecting the appropriate YOLO variant should consider deployment priorities, balancing real-time inference speed against maximum accuracy. Metrics derived from training logs provide reliable guidance to make informed choices based on application requirements.

### Detectron2 keypoint R-CNN (optimized configurations)

For comparison with YOLO, we further evaluated Detectron2 Keypoint R-CNN under optimized training configurations. Several candidate settings, including different learning rate schedules, batch sizes, and warmup iterations, were tested. The optimal configuration for each backbone (R50 and R101) was selected to represent the best trade-off between accuracy and training efficiency. All parameters not explicitly changed follow the default settings listed in Table 4, while Table 9 summarizes the optimized settings together with the resulting performance metrics and training statistics. Although some alternative settings achieved comparable results, we report only those configurations that yielded the best overall performance.

**Table 9:** Performance of Detectron2 under the best possible configurations. Training parameters are listed first for clarity, followed by performance metrics on the validation set (original dataset). All other parameters are consistent with those in Table 4.

Setting / Metric	Detectron2-R50 (tuned)	Detectron2-R101 (tuned)
**Training Parameters**		
Batch Size	4	4
Data Loader Workers	4	4
Evaluation Frequency	Every 1 epochs (70 iter)	Every 1 epochs (70 iter)
Warmup Factor	0.002	0.002
Warmup Iteration	1 (WARMUP_ITERS=1×70=70)	2 (WARMUP_ITERS=2×70=140)
Early Stopping Patience (same as in Table 4)	Enabled (patience = 10 epochs, i.e., 10 × 70 = 700 iters)	Enabled (patience = 10 epochs, i.e., 10 × 70 = 700 iters)
**Performance Metrics**		
mAP@0.5:0.95 (BBox)	56.59	58.93
mAP@0.5 (BBox)	93.11	94.07
mAP@0.5:0.95 (Keypoints)	37.81	38.15
mAP@0.5 (Keypoints)	58.55	59.23
Best Epoch	7	44
Total Training Time (h)	17 epochs (1190 iters): 0.632 h	54 epochs (3780 iters): 2.147 h

**Note:** Detectron2’s default scheduler is WarmupMultiStepLR. In our experiments, WarmupCosineLR consistently yielded more stable training and approximately a 2–3% increase in mAP@0.5:0.95 for keypoint detection; therefore, only those results are reported here.

**Key observations from the optimized Detectron2 backbones:**

- **Improved accuracy:** Compared to the original settings (see Table 4), and the baseline results reported in Table 6, both the tuned R50 and R101 backbones show improved performance for bounding boxes and keypoints, with the optimized results summarized in Table 9.
- **Training time:** Training time was also reduced for both backbones compared to the original configurations (see Table 4). The R50 backbone achieves competitive accuracy with shorter training time, while R101 provides slightly higher accuracy at the cost of substantially increased training time.
- **Overall optimization:** Overall, these optimized configurations balance accuracy and efficiency and provide a suitable basis for comparison with YOLO variants, although Detectron2 does not surpass YOLO models in keypoint accuracy.

### Robustness analysis (Optimized configurations)

We evaluate robustness across two sources of variability: random initialization and data augmentation. The first quantifies the stability of training, while the second assesses invariance to common image transformations. All models were trained under their optimized configurations, specifically those reported in Table 9, for Detectron2, to assess these robustness aspects, particularly for larger architectures.

### Robustness to random initialization

To quantify training stability under optimized hyperparameters, each model was trained across five independent runs with different random seeds.

For YOLOv8n-Pose and YOLOv11n-Pose, all stochastic augmentations were disabled, and training was conducted in a near-deterministic mode (fixed global seed and deterministic CUDA algorithms where supported). Under these conditions, both variants produced identical performance metrics across all runs, indicating zero run-to-run variance.

In contrast, Detectron2-based models (ResNet-50 and ResNet-101 backbones) exhibited measurable variability despite fixed global seeds. This nondeterminism arises from well-known sources in the training pipeline, including non-associative floating-point reductions on GPU, multi-worker data loading, mixed-precision arithmetic, and aspects of the evaluation procedure. Such behavior is widely documented in GPU-based deep learning frameworks, where full determinism remains difficult to achieve despite controlled settings [39].

Table 10 reports the mean ± standard deviation over the five runs for each model. YOLOv11n-Pose consistently achieved the highest performance among all models across all evaluated metrics, outperforming YOLOv8n-Pose and both Detectron2 variants.

**Table 10:** Performance of all models across five independent training runs on the test set (original dataset). Metrics are reported as mean ± standard deviation across five random seeds. Bold values indicate top performers.

Metric	YOLOv8n-Pose	YOLOv11n-Pose	Detectron2-R50	Detectron2-R101
**Bounding Box Metrics**				
mAP@0.5:0.95	**60.03 0.00**	59.60 0.00	55.90 1.05	54.10 1.56
mAP@0.5	92.19 ± 0.00	**94.97 ± 0.00**	92.86 ± 0.87	92.12 ± 1.68
**Keypoint Metrics**				
mAP@0.5:0.95 (OKS)	61.80 0.00	**62.20 0.00**	35.02 1.54	32.48 1.50
mAP@0.5 (OKS)	65.83 ± 0.00	**69.27 ± 0.00**	54.42 ± 0.42	51.46 ± 2.45

YOLO models, indicating stable and robust performance. While Detectron2 models showed higher variability, reflecting sensitivity to initialization in larger architectures, the observed standard deviations remained small relative to the absolute performance differences between models. Consequently, the comparative evaluation remains statistically sound, reliable, and reproducible.

### Augmentation robustness analysis

Building on the combined light augmentation results (see Table 7), we performed a targeted ablation study to assess the isolated impact of individual augmentation types: rotation, horizontal flipping, and random brightness/contrast, on keypoint localization performance. In these experiments, each augmentation was applied independently, doubling the training dataset size from 558 to 1116 images for each augmentation type, while validation and test sets remained unchanged to ensure fair comparison. Only keypoint-based metrics are reported, as bounding box performance remained largely stable across all augmentation variants and did not provide additional insight. This setup allows the effect of each augmentation on fine-grained anatomical keypoint localization to be evaluated without confounding influences from combined transformations.

Keypoint metrics on the test set are reported in Table 11. In this table, in addition to mAP@0.5:0.95 (OKS) and mAP@0.5 (OKS), we include the F1-score at OKS ≥ 0.5 to provide a more comprehensive evaluation.

**Table 11:** Effect of individual augmentation types on keypoint performance across all models on the test set (augmented dataset). Bold values indicate top performers.

Augmentation Type	YOLOv8n-Pose	YOLOv11n-Pose	Detectron2-R50	Detectron2-R101
**mAP@0.5:0.95 (OKS)**				
Random Rotate (p = 1.0)	63.89	**66.80**	33.90	32.00
Random Rotate (p = 1.0)	**57.04**	55.07	26.20	33.10
Horizontal Flip (p = 1.0)	**54.05**	46.75	16.50	18.70
Brightness/Contrast (±30%, p = 1.0)	**61.99**	60.00	34.20	29.80
**mAP@0.5 (OKS)**				
Random Rotate (p = 1.0)	60.86	**73.37**	51.20	49.80
Random Rotate (p = 1.0)	**61.46**	58.60	46.60	50.30
Horizontal Flip (p = 1.0)	**57.67**	51.97	31.70	39.40
Brightness/Contrast (±30%, p = 1.0)	**67.03**	56.63	53.20	47.10
**F1-score (OKS ≥ 0.5)**				
Random Rotate (p = 1.0)	68.39	**75.31**	67.94	65.56
Random Rotate (p = 1.0)	**63.27**	62.53	62.14	65.62
Horizontal Flip (p = 1.0)	**65.27**	59.30	51.69	56.38
Brightness/Contrast (±30%, p = 1.0)	**71.73**	68.32	66.45	64.54

While mAP reflects ranking performance across multiple OKS thresholds, it does not directly capture the balance between precision and recall at a fixed operating point. The F1-score addresses this by summarizing both detection reliability and localization accuracy under a defined OKS criterion.

This distinction is particularly important in medical pose estimation, where false positives and missed keypoints can have different practical implications, and dataset sizes are often limited. Including the F1-score therefore provides a more interpretable and task-relevant assessment of model performance, especially when comparing geometric (rotation, horizontal flipping) and photometric (brightness adjustment) augmentations that may affect precision and recall differently.

### Key observations on augmentation robustness:

- **Geometric vs. Photometric Transformations:** Geometric augmentations (rotation and horizontal flipping) consistently degrade keypoint localization accuracy across all evaluated architectures, whereas photometric changes such as brightness and contrast adjustments have only a minor impact on performance. In several cases, brightness and contrast perturbations yield competitive F1-scores relative to geometric transformations. This indicates that spatial transformations disrupt the learned anatomical structure more strongly than appearance-level variations, as photometric changes modify only pixel intensity distributions while preserving anatomical geometry.
- **Impact of Horizontal Flipping:** Horizontal flipping emerges as the most detrimental augmentation overall, producing substantial reductions in both mAP and F1-score, particularly for Detectron2-based models. This suggests that flipping disrupts learned spatial priors, especially the left–right anatomical relationships that are critical for accurate vertebral landmark localization.
- **Sensitivity to Rotations:** Small-angle rotations (±5°) lead to moderate performance degradation, whereas larger rotations (90°) produce more pronounced declines, particularly under the stricter *mAP*@0.5:0.95 (OKS) metric. This behavior indicates that large spatial transformations significantly alter the relative configuration of anatomical landmarks.
- **Robustness of YOLO Architectures:** YOLOv8n-Pose and YOLOv11n-Pose demonstrate greater robustness to spatial perturbations than the Detectron2 backbones (R50 and R101). Detectron2 models show increased sensitivity to geometric transformations, suggesting a stronger reliance on fixed spatial priors in two-stage architectures. These results indicate that single-stage pose estimation architectures may better tolerate geometric variability when trained on relatively limited medical imaging datasets.

Overall, this ablation study confirms that the performance degradation observed with combined augmentation (see Table 7) is primarily driven by geometric transformations rather than photometric ones. The negative impact is consistent across architectures, providing a clearer understanding of which augmentation strategies are detrimental to precise keypoint localization in medical pose estimation tasks.

## Discussion

This study demonstrates that model architecture, training settings, and data handling critically influence vertebrae keypoint detection performance, with notable trade-offs among accuracy, inference speed, and stability under random initialization and data perturbations. In particular, single-stage YOLO-based pose estimators exhibited superior reproducibility and robustness compared to larger two-stage Detectron2 architectures, especially on limited medical datasets. These findings underscore that, for clinical translation in applications such as automated spinal analysis and deformity assessment, model selection should prioritize not only peak accuracy but also reliable and consistent performance across variable conditions.

### Training and batch size

In our experiments, batch size had a strong influence on training time. For large models such as Detectron2 (R50, R101) and YOLOv11x, using a batch size of 8 with eight data loader workers led to very slow training, mainly due to CPU overload from parallel data loading and I/O bottlenecks. Reducing both the number of workers and the batch size to 4 improved performance. In our hardware-constrained environment, the smaller batch size reduced CPU contention and data loading overhead, which improved overall training efficiency and more consistent GPU utilization.

These findings suggest that, for heavy models, careful tuning of batch size and data loader workers is essential, with batch size often being the dominant factor in training speed. Optimal settings still depend on GPU memory, CPU capacity, storage speed, and the overall system configuration.

Even after adjusting parameters, Detectron2 was still slower and less consistent than YOLO models. One contributing factor is the higher number of trainable parameters in Detectron2, which increases computational demand and prolongs training. Table 9 shows that careful tuning improved Detectron2 performance, and we also observed that the choice of learning rate scheduler affected stability: using WarmupCosineLR provided smoother convergence and a small improvement in keypoint accuracy. Nevertheless, YOLO models achieved higher overall performance while requiring less training time.

### Early stopping and patience

For YOLO models, setting the early stopping patience to 20 epochs yielded better results than 10, likely because these models are smaller and benefited from additional training epochs. For Detectron2, a patience equivalent to 10 epochs worked best, as increasing it did not improve results. These details are omitted from the tables for brevity and focus, but highlight how the choice of patience parameter can affect convergence speed and final performance differently across architectures.

### Model size and performance

Increasing model size did not consistently improve performance. Detectron2 R101 provided only marginal gains over R50, while incurring longer training and inference times, and neither model reliably detected all keypoints. The frequent duplicate or missing keypoint predictions observed in Detectron2-based models are consistent with known limitations of two-stage detection-based pipelines in spinal landmark estimation, where anatomically repetitive vertebrae and the absence of explicit sequential constraints make it difficult to balance precision and recall, leading to false positives and false negatives despite post-processing such as non-maximum suppression [40]. Similar behaviors have been reported in prior spine keypoint studies using two-stage detectors, including Faster R-CNN variants [41]. In contrast, the single-stage YOLO-based pose estimators evaluated in our study impose a more constrained prediction structure, which reduces redundancy and improves spatial consistency for repetitive anatomical landmarks.

YOLOv11n-Pose performed better than YOLOv8n-Pose on keypoints without slowing inference, achieving the highest overall mean average precision (mAP), though YOLOv8n-Pose remained competitive across several metrics (see Tables 6 and 7). The consistent superiority of YOLOv11n over YOLOv8n in our controlled deterministic setting suggests architectural improvements in the *v11* backbone and neck contribute meaningfully to this task, beyond what is typically observed in general-purpose COCO benchmarks. These trade-offs highlight the importance of selecting models based on application constraints, particularly considering that all models were originally trained for 17 keypoints in human pose datasets.

We also evaluated other available YOLO variants (YOLOv8 and YOLOv11 Pose-families). In general, performance differences were minor, with some YOLOv8 variants performing slightly better or worse than YOLOv8n-Pose, and other YOLOv11 variants showing similar performance compared to YOLOv11n-Pose. An exception was observed for YOLOv8l-Pose and YOLOv11l-Pose, which performed better than their respective families. Notably, YOLOv8l-Pose achieved the highest overall accuracy despite being an older and smaller architecture, indicating that larger or newer architectures do not necessarily yield better performance for this task under the dataset size and training conditions used.

### Model stability

Model stability emerged as a critical consideration for vertebrae keypoint detection, particularly given the limited size of medical imaging datasets. To ensure reproducibility, all experiments were conducted with a fixed global random seed controlling weight initialization and data shuffling. Under these minimized randomness conditions, lightweight single-stage YOLO-based models demonstrated highly consistent performance across training runs, whereas larger two-stage architectures (e.g., Detectron2 variants) exhibited substantially greater variability (see Table 10). The observed run-to-run variability in Detectron2 models highlights that deterministic training is difficult to achieve in two-stage pipelines, even with fixed seeds and deterministic CUDA settings, which may limit their reproducibility and reliability in clinical contexts.

This increased sensitivity in two-stage models is likely related to their higher model capacity and more complex training pipelines, which require careful optimization and are more susceptible to training variability in limited-data biomedical settings. Prior studies have demonstrated that parameter-heavy deep learning models trained on relatively small medical imaging datasets exhibit increased performance variance and diminishing returns with growing complexity, largely due to stochastic factors such as weight initialization, data sampling, and optimizer dynamics [42]. In addition, the multi-stage refinement process in two-stage detectors may further amplify such variability through cascading error propagation and sensitivity to intermediate predictions. In contrast, single-stage YOLO-based estimators adopt a simpler, direct prediction structure, which reduces training complexity and contributes to improved robustness and consistency in our experiments.

These findings underscore that model selection for medical applications should prioritize not only peak accuracy but also robustness and reproducibility. Using default training settings with fixed seeds provides a reliable baseline before task-specific tuning, helping mitigate risks of performance drift in resource-constrained or data-limited settings.

### Data augmentation effects on pose estimation

Data augmentation had a nuanced effect on pose estimation. Light augmentations were sometimes beneficial, but moderate or aggressive transformations such as rotation, scaling, or translation often reduced performance. In some cases, keypoints were displaced outside the image boundaries, compromising label accuracy. Consequently, only light augmentations were used to preserve anatomical structure while introducing limited variation (see Tables 5 and 11). In particular, geometry-based transformations disrupted spatial correspondence between anatomical landmarks, whereas appearance-based perturbations that preserved structure were generally better tolerated. This observation is consistent with prior spine radiograph landmark studies, which emphasize preserving anatomical geometry and vertebral alignment during training to ensure reliable localization and angle estimation [37,43].

Single-stage YOLO-based models showed greater robustness to these perturbations, likely due to their direct, end-to-end prediction approach. Two-stage architectures such as Detectron2 rely on sequential refinement stages and place greater emphasis on learned spatial priors, which can make them more sensitive to geometric transformations.

Taken together, these findings demonstrate that the negative impact of data augmentation is both task-specific (tied to the highly structured anatomy of the spine) and model-specific (driven by fundamental differences in how architectures encode and enforce spatial constraints). Maintaining spatial integrity appears to be more important than increasing visual diversity for medical pose estimation.

### Built-in augmentations in YOLO pose models

YOLO Pose models include additional built-in augmentations such as Mosaic, MixUp, and color jitter. In our experiments, these predefined augmentations produced inconsistent effects on performance: under default stochastic settings, YOLOv8n-Pose occasionally outperformed YOLOv11n-Pose, reflecting variability introduced by randomness and augmentation. To ensure a uniform comparison across models, all automated augmentations were disabled. Under these deterministic conditions (with randomness disabled), YOLOv11n-Pose consistently outperformed YOLOv8n-Pose, even on the same manually augmented dataset (see Table 11). Moreover, comparing the original and augmented datasets revealed a slight accuracy decrease for YOLOv8n-Pose and Detectron2, while YOLOv11n-Pose remained stable, further indicating that the performance advantage of YOLOv11n-Pose is robust and architectural in nature.

### Evaluation metrics

Both standard COCO-style metrics and custom criteria were used to capture vertebra detection and keypoint localization performance. Relaxed thresholds (e.g., mAP@0.5 or lenient distance cutoffs) can yield high scores but may overestimate true accuracy, whereas stricter benchmarks reflect clinically meaningful precision. This combination provides a balanced and interpretable assessment, illustrating both generalization and practical utility in medical imaging.

### Limitations of the study

While our experiments provide a detailed comparison of YOLO and Detectron2 architectures for vertebrae keypoint detection, several limitations should be noted. The dataset comprised only lateral lumbar X-ray images (698 images), which may limit generalizability to other spinal regions, imaging modalities (e.g., MRI, CT), or patient populations. Although evaluation was performed across all YOLO model scales (see Table 8), the Nano variants were prioritized for clinical efficiency. The observed performance decline in Extra-Large YOLO variants and Detectron2 architectures suggests that the current dataset size is a limiting factor for these high-parameter models, indicating a performance-to-parameter mismatch for specialized medical datasets of this scale.

Using four keypoints per vertebra may not fully capture anatomical complexity. Occlusions and anatomical variability in clinical images are likely to reduce keypoint reliability, which should be considered when interpreting results. Additionally, our experiments did not investigate multi-modal or cross-modality generalization, which is critical for clinical applications where imaging protocols vary. Future studies could explore domain adaptation or multi-modal training to improve robustness across X-ray, MRI, and CT data.

Nevertheless, the comparative trends observed between YOLO and Detectron2 architectures, particularly the superior robustness and reproducibility of YOLO, are expected to hold under similar imaging conditions, although absolute performance may vary.

### Future work

Future research should focus on enhancing accuracy and generalization by leveraging larger, more diverse datasets and integrating semi-supervised or weakly-supervised learning to reduce annotation dependency. Automated hyperparameter optimization and neural architecture search could further improve model performance efficiently.

Beyond 2D, extending investigations to 3D imaging and multi-view datasets can enhance clinical relevance by capturing richer anatomical context. Domain-specific augmentations tailored to spinal imagery can help models generalize across patient populations and imaging conditions. Refining keypoint heatmap regression and incorporating advanced architectures, such as graph neural networks or transformer-based models, could significantly improve the precision and reliability of automatic vertebrae detection and pose estimation in clinical applications.

Additionally, future studies could conduct systematic comparisons of multiple model architectures on standardized vertebrae and spine datasets, helping clinicians identify the best-performing and fastest models for specific clinical tasks. Extending evaluations to additional pose estimation architectures, such as HRNet, OpenPose, or spine-specific graph-based methods across diverse dataset conditions, would provide deeper insights and better guide practical clinical adoption. Further investigations should explore model behavior under varying dataset sizes and characteristics, optimal hyperparameter tuning for different resource constraints, robustness to occlusions, standardized inference times normalized across hardware and batch sizes, as well as rigorous evaluation via cross-validation or on larger external datasets. These efforts would enhance generalizability, facilitate efficient model selection, and support reliable deployment in real-world clinical settings.

### Clinical relevance

Our results have direct implications for vertebrae keypoint detection in clinical settings. YOLO-based models strike a favorable balance between speed and accuracy, enabling near real-time applications such as automated X-ray analysis, surgical planning, or deformity assessment (e.g., scoliosis via Cobb angle measurement). Importantly, their high stability and consistent, non-duplicated keypoint localization reduce the risk of error propagation into downstream measurements such as intervertebral alignment or angular estimation, which is critical for reliable clinical interpretation. These findings underscore the value of prioritizing both accuracy and computational efficiency when selecting models for practical clinical deployment.

## Conclusion

This study presented a controlled comparative evaluation of pose-based deep learning models (YOLOv8n-Pose, YOLOv11n-Pose, and Detectron2 with ResNet-50 and ResNet-101 backbones) for vertebrae detection and four-keypoint localization. Using a standardized dataset, consistent training configurations, and evaluation under both original and augmented conditions, we investigated the balance between accuracy, robustness, and computational efficiency across architectures.

The results demonstrate that YOLOv11n-Pose achieves the best balance between accuracy and inference speed, offering consistent keypoint detection with minimal degradation under augmentation. YOLOv8n-Pose provides very fast inference with competitive accuracy, making it a strong choice for resource-limited or real-time applications. In contrast, Detectron2-based models delivered lower keypoint accuracy, slower inference, and frequent issues with missing (due to duplicate predictions), despite careful hyperparameter tuning.

Additional experiments with larger YOLO variants revealed that YOLOv8l-Pose achieved the highest overall accuracy across bounding box and keypoint tasks, though at the cost of increased training time. Importantly, these findings suggest that larger model size or newer architectures alone do not guarantee superior performance in medical imaging tasks; instead, performance depends on the balance between dataset characteristics, annotation quality, and computational constraints.

Overall, these findings provide practical guidance for selecting vertebrae keypoint detection models based on the trade-offs between accuracy, speed, and available computational resources.
